# Patient Age and the Prognosis of Idiopathic Membranous Nephropathy

**DOI:** 10.1371/journal.pone.0110376

**Published:** 2014-10-20

**Authors:** Makoto Yamaguchi, Masahiko Ando, Ryohei Yamamoto, Shinichi Akiyama, Sawako Kato, Takayuki Katsuno, Tomoki Kosugi, Waichi Sato, Naotake Tsuboi, Yoshinari Yasuda, Masashi Mizuno, Yasuhiko Ito, Seiichi Matsuo, Shoichi Maruyama

**Affiliations:** 1 Department of Nephrology, Nagoya University Graduate School of Medicine, Nagoya, Japan; 2 Center for Advanced Medicine and Clinical Research, Nagoya University Hospital, Nagoya, Japan; 3 Department of Geriatric Medicine and Nephrology, Osaka University Graduate School of Medicine, Suita, Japan; Mario Negri Institute for Pharmacological Research and Azienda Ospedaliera Ospedali Riuniti di Bergamo, Italy

## Abstract

**Background:**

Idiopathic membranous nephropathy (IMN) is increasingly seen in older patients. However, differences in disease presentation and outcomes between older and younger IMN patients remain controversial. We compared patient characteristics between younger and older IMN patients.

**Methods:**

We recruited 171 Japanese patients with IMN, including 90 (52.6%) patients <65 years old, 40 (23.4%) patients 65–70 years, and 41 (24.0%) patients ≥71 years. Clinical characteristics and outcomes were compared between younger and older IMN patients.

**Results:**

During a median observation period of 37 months, 103 (60.2%) patients achieved complete proteinuria remission, which was not significantly associated with patient age (*P* = 0.831). However, 13 (7.6%) patients were hospitalized because of infection. Multivariate Cox proportional hazards models identified older age [adjusted hazard ratio (HR) = 3.11, 95% confidence interval (CI): 1.45–7.49, per 10 years; *P* = 0.003], prednisolone use (adjusted HR = 11.8, 95% CI: 1.59–242.5; *P* = 0.014), and cyclosporine used in combination with prednisolone (adjusted HR = 10.3, 95% CI: 1.59–204.4; *P* = 0.012) as significant predictors of infection. A <25% decrease in proteinuria at 1 month after immunosuppressive therapy initiation also predicted infection (adjusted HR = 6.72, 95% CI: 1.51–37.8; *P* = 0.012).

**Conclusions:**

Younger and older IMN patients had similar renal outcomes. However, older patients were more likely to develop infection when using immunosuppressants. Patients with a poor response in the first month following the initiation of immunosuppressive therapy should be carefully monitored for infection and may require a faster prednisolone taper.

## Introduction

In Japan, the elderly population (i.e., those aged 65 and over) comprised 24.1% of the total population as of October 2012, a figure that is expected to increase to 39.9% by 2060 [1]. With increases in life expectancy, greater numbers of elderly patients with chronic kidney diseases are surviving longer. Membranous nephropathy (MN) is the most important cause of nephrotic syndrome in elderly patients [2], [3]. The incidence of MN is higher in the elderly than in younger adults. Although little information is available regarding the natural course of MN in elderly patients, Zent et al. [4] reported similar clinical presentations between older and younger patients. However, clinical information such as the prevalence of leg edema or pleural effusion was not included. Thus, the clinical severity associated with nephrotic syndrome could not be adequately evaluated.

Most randomized trials of immunosuppressive therapy for MN included few, if any, patients older than 65 [5]–[9], and only a few retrospective studies and case series specifically reported immunosuppressive therapy outcomes [4], [10], [11]. Consequently, the optimal immunosuppressive regimen for elderly idiopathic membranous nephropathy (IMN) patients remains controversial.

Based on our clinical experience, our impression is that compared to younger IMN patients, older patients have more severe symptoms associated with nephrotic syndrome and are more susceptible to infection. It can therefore be difficult to weigh the risks and benefits of initiating immunosuppressive therapy, especially among elderly patients.

To understand the clinical characteristics of elderly IMN patients and identify patients at high risk for infection, we conducted a retrospective multicenter observational cohort study that was organized as part of the Nagoya Nephrotic Syndrome Cohort Study (N-NSCS), a study based in 10 major nephrology centers in Nagoya, Japan.

## Subjects and Methods

### Study Population and Data Sources

Participants in the present study were included in our previous multicenter retrospective cohort study, N-NSCS, which identified cigarette smoking as a risk factor for kidney dysfunction for IMN [12]. The study design of N-NSCS was described in detail elsewhere [12]. Briefly, this study included patients older than 18 years who were diagnosed with MN based on kidney biopsy results at Nagoya University, Chubu Rosai Hospital, Japanese Red Cross Nagoya Daiichi Hospital, Tsushima City Hospital, Kasugai Municipal Hospital, Nagoya Kyoritsu Hospital, Anjo Kosei Hospital, Ichinomiya Municipal Hospital, Handa City Hospital, or Tosei General Hospital between January 2003 and December 2012. Out of 272 identified MN patients, we excluded those with conditions generally considered to cause secondary MN [10]. Furthermore, we excluded seven patients (3.9%) because of loss to follow-up (n = 6) and missing data (n = 1). Ultimately, 171 (62.9%) IMN patients were enrolled and followed up until September 2013.

Our study was conducted by using linkable anonymous data set. No informed consent was obtained. The study protocol and consent procedure were approved by the ethics committees of Nagoya University, Chubu Rosai Hospital, Japanese Red Cross Nagoya Daiichi Hospital, Tsushima City Hospital, Kasugai Municipal Hospital, Nagoya Kyoritsu Hospital, Anjo Kosei Hospital, Ichinomiya Municipal Hospital, Handa City Hospital, and Tosei General Hospital.

### Data Collection

Baseline characteristics were collected retrospectively from patients’ medical records. Clinical characteristics at the time of kidney biopsy were considered to represent baseline if the patient had not received immunosuppressive therapy or received immunosuppressive therapy only after a kidney biopsy. For patients who received immunosuppressive therapy before biopsy, the clinical characteristics at the time of initiating immunosuppressive therapy were used as baseline. Baseline characteristics included age, gender, body mass index, systolic and diastolic blood pressure, serum total cholesterol, serum creatinine, glomerular filtration rate [GFR; estimated using the equation recently developed by the Japanese Society of Nephrology: eGFR (mL/min/1.73 m^2^) = 194×Scr^−1.094^×Age^−0.287^×0.739 (if female) (13)], serum albumin, 24-hour urinary protein excretion or urinary protein/creatinine ratio, smoking status, antihypertensive drug use, and initial use of corticosteroids and/or other immunosuppressive agents.

Antihypertensive drugs used in this cohort included angiotensin-converting enzyme (ACE) inhibitors or angiotensin II receptor blockers (ARB), calcium channel blockers, β-blockers, and thiazides. Information regarding therapeutic interventions was also collected, including the use of ACE inhibitors/ARBs and corticosteroids or other immunosuppressive agents that were prescribed during the observation period.

Nephrotic syndrome was defined as a urinary protein excretion ≥3.5 g/day (or a urinary protein/creatinine ratio ≥3.5) and a serum albumin level <3.0 mg/dL.

Complete remission (CR) from proteinuria was defined as a urinary protein excretion <0.3 g/day, a urinary protein/creatinine ratio <0.3, and/or a negative/trace result for urinary protein on a dipstick test. Partial remission (PR) from proteinuria was defined as a urinary protein excretion <3.5 g/day and a urinary protein/creatinine ratio <3.5. Relapse was defined as a urinary protein excretion ≥1.0 g/day, a urinary protein/creatinine ratio ≥1.0, or a urinary protein dipstick result ≥2+ on at least two occasions after CR had been achieved.

The rate of eGFR decline per year (mL/min per 1.73 m^2^/year) was determined by plotting eGFR against the observation time.

The anonymous data set is available in the **Table S7** **in File S2**.

### Outcomes

Our primary outcome was the first CR. Secondary outcomes were hospitalization due to infection and a 30% decline in the eGFR before end-stage renal disease (ESRD). Patients who died before achieving either outcome were censored at the time of death. The eGFR was measured as required for each patient at 1–3 month intervals. Patients were followed up until September 2013 and censored at the time of their death before ESRD or as of their last serum creatinine measurement before September 2013.

### Statistical Analyses

We stratified patients into three age categories: <65, 65–70, and ≥71 years old. Clinical characteristics were compared between these three groups using a Wilcoxon rank-sum test or Fisher’s exact test. To determine predictors independently associated with each outcome, potential covariates were assessed using a log-rank test and/or univariate and multivariate Cox proportional hazards (CPH) models. For continuous variables, a Wilcoxon rank-sum test was used to assess the significance of inter-group differences. Results for categorical variables were expressed as percentages and compared by using Fisher’s exact test. The cumulative probabilities of achieving a first CR and hospitalization due to infection were determined using the Kaplan-Meier method and log-rank tests. Predictors of these outcomes were identified using univariate and multivariate CPH models. The proportional hazards assumption for covariates was tested using scaled Schoenfeld residuals. A−2 log likelihood value for fitting a model with all explanatory variables was determined for individual CPH models that included 25%, 50%, or 75% decreases in proteinuria in the first month after initial immunosuppressive therapy, and was used to compare the performance of these three models. A likelihood ratio test was used to determine whether the fit of a model that included a 25% decrease rate was the best model for the present study. The trend in the outcome with respect to the decreases in proteinuria in the first month after initial immunosuppressive therapy was examined statistically by scoring ≥50% decrease as 0 and 25–50% decrease, 0–25% decrease, and exacerbation as 1, 2, and 3, respectively; the resulting scores were then included in the regression model. Least squares mean±95% confidence intervals for urinary protein and eGFR during follow-up period were compared between three age categories using linear mixed-effect models.

The level of statistical significance was set at *P*<0.05. All statistical analyses were performed using JMP version 10.0.0 (SAS Institute, Cary, NC, USA; www.jmp.com), SAS version 9.4 (SAS Institute, Cary, NC; www.sas.com), and STATA version 13.0 (STATA Corp, www.stata.com).

## Results

### Clinical Characteristics

A total of 171 patients were diagnosed with IMN after appropriate clinical and laboratory screening for secondary causes. Baseline characteristics stratified by the three age categories are shown in Table 1.

**Table 1:** Baseline characteristics of 171 IMN patients.

	<65 years	65–70 years	≥71 years	*P*-value
Number	90	40	41	
Baseline characteristics				
Age (years)	57 (50–62)	68 (66–69)	75 (74–78)	
Male [n (%)]	63 (70.0)	28 (70.0)	27 (65.9)	0.882
Body mass index (kg/m^2^)	23.2 (21.2–25.8)	23.2 (22.0–25.7)	22.6 (21.1–24.6)	0.440
Systolic blood pressure (mmHg)	129 (120–140)	135 (125–158)	138 (122–147)	0.028
Diastolic blood pressure (mmHg)	77 (70–86)	80 (70–86)	76 (70–84)	0.502
Serum creatinine (mg/dL)	0.77 (0.68–0.90)	0.80 (0.68–0.99)	0.95 (0.7–1.2)	0.011
eGFR (mL/min/1.73 m^2^)	81 (70–97)	73 (58–86)	59 (46–81)	<0.001
Serum albumin (g/dL)	2.8 (2.1–3.5)	2.5 (2.0–3.1)	2.3 (1.9–2.8)	0.014
Urinary protein (g/day)	4.2 (2.6–7.0)	3.6 (2.6–7.6)	5.1 (3.3–7.7)	0.275
Urinary protein >3.5 (g/day) [n (%)]	29 (32.2)	17 (42.5)	11 (26.8)	0.310
Total cholesterol (mg/dL)	279 (229–382)	284 (239–400)	297 (249–367)	0.859
Leg edema [n (%)]	64 (71.1)	29 (72.5)	38 (92.7)	0.020
Pleural effusion [n (%)]	12 (13.3)	8 (20.0)	13 (31.7)	0.048
Treatment				
ACE inhibitor or ARB therapy [n (%)]	80 (88.9)	39 (97.5)	47 (90.2)	0.268
Immunosuppressive therapy				0.893
No immunosuppressants	36 (40.0)	17 (42.5)	17 (41.5)	
Prednisolone [n (%)]	17 (18.9)	10 (25.0)	8 (19.5)	
Prednisolone+Cyclosporine [n (%)]	37 (41.1)	13 (32.5)	16 (39.0)	
Observational period (months)	44 (20–86)	31 (15–63)	25 (11–54)	0.021

NOTE: Median (interquartile range), Conversion factors for units: SCr in mg/dL to µmol/L, ×88.4; eGFR (mL/min/1.73 m2) = 194×Scr^−1.094^×Age^−0.287^×0.739 (if female), total cholesterol in mg/dL to mmol/L, ×0.02586.

Abbreviations: IMN, idiopathic membranous nephropathy; eGFR, estimated glomerular filtration rate; ACE inhibitor/ARB, angiotensin-converting enzyme inhibitor/angiotensin receptor blocker.

The cohort included 90 (52.6%) patients <65 years old, 40 (23.4%) aged 65–70 years, and 41 (24.0%) ≥71 years old. Compared with younger patients, older patients had lower serum albumin levels (*P* = 0.014), lower eGFRs (*P*<0.001), higher serum creatinine levels (*P* = 0.011), higher systolic blood pressure (*P* = 0.028), and a higher prevalence of leg edema and pleural effusion (*P* = 0.020 and *P* = 0.048, respectively). This suggested a greater severity of symptoms associated with nephrotic syndrome in older patients than in younger patients. The prevalence of pleural effusion was determined by chest radiography results at the time of kidney biopsy. No trends were found for initial immunosuppressive therapy among the different age categories. The observation periods were shorter in the elderly group (*P* = 0.021).

### Treatment During the Observation Period

During follow-up, 156 (91.2%) patients used ACE inhibitors or ARBs. Patients were divided into three groups according to the type of treatment they received during the observation period: (1) a prednisolone group comprising 35 patients (20.5%) who received prednisolone alone; (2) a cyclosporine group comprising 66 patients (38.6%) who received prednisolone and cyclosporine; and (3) a supportive therapy group comprising 70 patients (40.9%) who did not receive prednisolone or other immunosuppressive drugs. One patient in the cyclosporine group (0.6%) developed a 50% serum creatinine increase over baseline and was prescribed mizoribine.

The median initial prednisolone dose was 30 mg (interquartile range: 20–40 mg/day) and was tapered according to treatment response. Most patients in the cyclosporine group were started on cyclosporine at 1–2 mg/kg and prednisolone at 0.4–0.6 mg/kg. Cyclosporine dosing was modified by monitoring whole-blood trough levels, while prednisolone was tapered according to treatment response. The time from kidney biopsy to immunosuppressive therapy initiation was 0.7 months (interquartile range: 0.3–3.3 months).

### Outcomes

#### Primary outcome: first complete remission

Outcome data are shown in Table 2. The median observation period for the entire cohort was 37 months (interquartile range: 15–72 months). CR from proteinuria was achieved by 103 (60.2%) patients. The mean time to CR was 14 months (interquartile range: 6–25 months).

**Table 2:** Outcomes of 171 IMN patients.

	<65 years	65–70 years	≥71 years	P-value
Number	90	40	41	
30% reduction in eGFR [n (%)]	17 (18.9)	9 (22.5)	11 (26.8)	0.591
Decline in eGFR (mL/min per1.73 m^2^ per year)	2.37 (0.25–7.43)	3.06 (−2.17–8.33)	4.10 (−0.58–8.31)	0.956
ESRD [n (%)]	1 (1.1)	0 (0.0)	1 (2.4)	1.000
Death [n (%)]	1 (1.1)	3 (7.5)	7 (17.1)	0.003
Death due to infection [n (%)]	0 (0.0)	1 (2.5)	6 (14.6)	<0.001
Hospitalization due to infection [n (%)]	2 (2.2)	3 (7.5)	8 (19.5)	0.003
Hospitalization due to cardiovascular disease [n (%)]	1 (1.1)	0 (0.0)	2 (4.9)	0.197
Venous thrombotic events [n (%)]	0 (0.0)	0 (0.0)	0 (0.0)	1.000
Malignancy [n (%)]	2 (2.2)	2 (5.0)	1 (2.4)	0.671
Steroid psychosis [n (%)]	1 (1.1)	1 (2.5)	1 (2.4)	0.756
Use of antidiabetic agents [n (%)]	8 (8.9)	3 (7.5)	2 (4.9)	0.724
Aseptic osteonecrosis with surgical treatment [n (%)]	0 (0.0)	0 (0.0)	0 (0.0)	1.000
Remission				
Complete remission [n (%)]	56 (62.2)	26 (65.0)	21 (51.2)	0.383
Partial remission [n (%)]	81 (90.0)	36 (90.0)	33 (80.5)	0.270
Relapse [n (%)]	17 (24.3)	2 (6.7)	7 (21.9)	0.120

NOTE: Median (interquartile range), Conversion factors for units: SCr in mg/dL to µmol/L, ×88.4; eGFR (mL/min/1.73 m2) = 194×Scr^−1.094^×Age^−0.287^×0.739 (if female), total cholesterol in mg/dL to mmol/L, ×0.02586.

Abbreviations: IMN, idiopathic membranous nephropathy; eGFR, estimated glomerular filtration rate; ESRD, end-stage renal disease.

**Figure 1:**
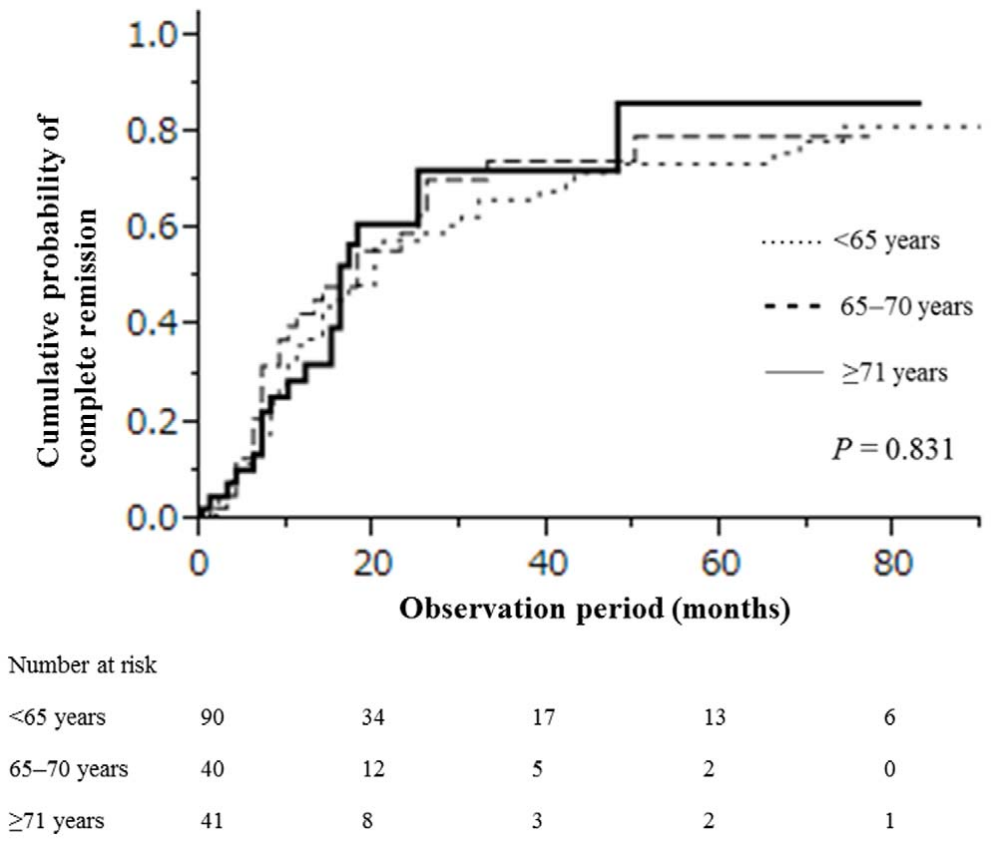
Cumulative probability of complete remission in 171 IMN patients stratified by age.

The cumulative probabilities of achieving a CR within 1, 5, and 10 years were, respectively, 0.37, 0.74, and 0.81 for patients <65 years old; 0.45, 0.79, and 0.79 for those 65–70 years old; and 0.32, 0.86, and 0.86 for those ≥71 years old. There were no significant differences in CR from proteinuria according to patient age (*P* = 0.831; **Figure 1**).

Furthermore, the changes in average proteinuria over time did not differ among the various age groups (*P* = 0.458; **Figure S1**).

Among patients who achieved a first remission, 26 (25.2%) relapsed at least once.

#### Secondary outcomes: hospitalization due to infection and a 30% decline in eGFR level

During follow-up, 37 (21.6%) patients developed a 30% decline in eGFR before ESRD. In all 37 patients, eGFR levels did not improve by the last follow-up visit.

Although there were no significant differences according to patient age in 30% eGFR declines and eGFR decline rates, the average eGFR over time in patients <65 years old was significantly higher than that in ≥71 years old (*P* = 0.046; **Figure S2**).

**Figure 2:**
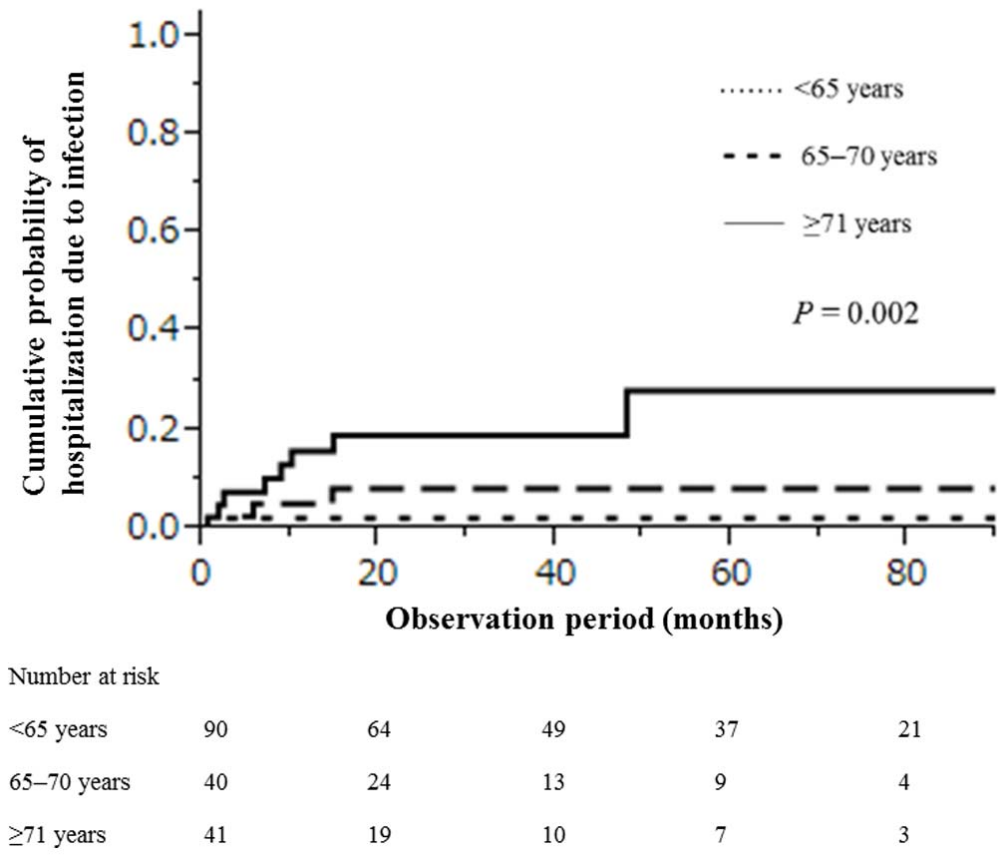
Cumulative probability of hospitalization due to infection in 171 IMN patients stratified by age.

Infection caused 13 (7.6%) individual patient hospitalizations and 7 (4.1%) deaths. The older age categories were significantly associated with hospitalization and death due to infection (*P* = 0.003). The cumulative probabilities of hospitalization due to infection within 1, 5, and 10 years were, respectively, 0.02, 0.02, and 0.02 for those <65 years old; 0.05, 0.08, and 0.08 for those 65–70 years old; and 0.16, 0.28, and 0.28 for those ≥71 years old. There were significant differences in hospitalization due to infection according to patient age (*P* = 0.002; **Figure 2**).

Causes of infection were tuberculosis pleuritis (n = 1), bacterial pneumonia (n = 7), *Pneumocystis jiroveci* pneumonia (n = 1), vertebral osteomyelitis (n = 1), methicillin-resistant *Staphylococcus aureus* bacteremia (n = 1), fungemia (n = 1), and pyelonephritis (n = 1).

After excluding seven patients who died because of infection, the remaining causes of death were acute subdural hematoma (n = 1), traffic accident (n = 1), sudden death (n = 1), and intestinal bleeding (n = 1). Malignancy occurred in five (2.9%) patients, whose diagnoses included esophageal cancer (n = 1), stomach cancer (n = 1), colon cancer (n = 1), prostate cancer (n = 1), and malignant lymphoma (n = 1). Only three (1.8%) patients were hospitalized for cardiovascular disease, and no patient had a venous thromboembolic event.

### Treatment and clinical characteristics: comparison of patients in different treatment groups

Clinical characteristics of the three groups including (1) the prednisolone monotherapy group, (2) the combined cyclosporine group, and (3) the supportive therapy group are shown in **Tables S1** **and S2** **in File S1**. The serum albumin level in the supportive therapy group was significantly higher than that in the immunosuppressive therapy groups (prednisolone monotherapy and combined cyclosporine groups) (*P<*0.001), and the urinary protein level in the supportive therapy group was significantly lower than that in the immunosuppressive therapy group (*P*<0.001). With respect to outcomes, the proportion of patients achieving a 30% eGFR decrease was not significantly different among the three groups (*P* = 0.591), whereas the proportion of patients achieving CR and the rate of hospitalization due to infection were significantly higher in the immunosuppressive therapy groups than the supportive group (*P* = 0.031 and *P* = 0.032, respectively).

### Predictors of First Complete Remission

Univariate CPH models showed that a lower serum creatinine level, initial use of prednisolone monotherapy, and initial use of cyclosporine in combination with prednisolone were significantly associated with a first CR from proteinuria (**Table 3**). After adjusting for clinically relevant factors, the initial use of prednisolone monotherapy [adjusted hazard ratio (HR) = 1.78, 95% confidence interval (CI): 1.01–3.10; *P* = 0.045) and cyclosporine in combination with prednisolone (adjusted HR = 1.95. 95% CI: 1.16–3.26; *P* = 0.011) remained as significant predictors of a first CR from proteinuria.

**Table 3:** Predictors of first CR.

	Univariate model		Multivariate model	
	HR (95% CI)	*P*-value	HR (95% CI)	*P*-value
Age (per 10 years)	0.99 (0.83–1.19)	0.886	0.98 (0.79–1.23)	0.759
Male (versus female)	0.69 (0.46–1.04)	0.075	0.77 (0.47–1.27)	0.302
Systolic blood pressure (per 10 mmHg)	1.00 (0.91–1.09)	0.959	1.06 (0.96–1.17)	0.276
Serum albumin (per 1.0 g/dL)	0.86 (0.67–1.11)	0.249	1.04 (0.74–1.45)	0.818
Serum creatinine (per 1.0 mg/dL)	0.47 (0.21–0.96)	0.037	0.25 (0.03–1.68)	0.163
Urinary protein excretion (per 1.0 g/day)	1.01 (0.95–1.06)	0.730	1.16 (0.29–4.18)	0.823
ACE inhibitor or ARB therapy	0.66 (0.44–1.02)	0.061	0.56 (0.26–1.31)	0.170
Immunosuppressive treatmentduring follow-up period				
No immunosuppressive agents	Reference		Reference	
Prednisolone	1.76 (1.00–2.96)	0.048	1.78 (1.01–3.10)	0.045
Prednisolone+cyclosporine	2.18 (1.41–3.36)	<0.001	1.95 (1.16–3.26)	0.011

NOTE: HR, hazard ratio; CI, confidence interval.

Data are the HR, 95% CI, and *P*-value from Cox proportional hazard regression analyses.

Adjusted for baseline characteristics (age, sex, systolic pressure, serum albumin level, serum creatinine level, urinary protein, use of immunosuppressive therapy).

Abbreviations: CR, complete remission; IMN, idiopathic membranous nephropathy.

### Predictors of Infection

A total of 13 (7.6%) patients had at least one infection that required hospitalization. Of these, 12 (92.3%) received immunosuppressive therapy before developing an infection. The median time from immunosuppressive therapy initiation to first hospitalization due to infection was 3 months (interquartile range: 1–5 months). When examining all hospitalizations due to infections, univariate analyses identified several statistically significant predictors of increased infection risk: older age, higher serum creatinine levels, and immunosuppressive therapy use, namely prednisolone monotherapy and cyclosporine combination therapy (**Table 4**).

**Table 4:** Predictors of hospitalization due to infection (n = 171).

	Univariate model		Multivariate model	
	HR (95% CI)	*P*-value	HR (95% CI)	*P*-value
Age (per 10 years)	3.07 (1.56–6.58)	<0.001	3.11 (1.45–7.49)	0.003
Male (versus female)	1.49 (0.45–6.63)	0.533	1.34 (0.37–6.29)	0.666
Serum albumin (per 1.0 g/dL)	0.73 (0.33–1.50)	0.394	1.24 (0.47–3.21)	0.662
Serum creatinine (per 1.0 mg/dL)	5.27 (1.81–12.5)	0.004	2.62 (0.83–7.70)	0.098
Urinary protein excretion (per 1.0 g/day)	0.96 (0.79–1.12)	0.613	0.92 (0.71–1.13)	0.468
Immunosuppressive treatmentduring follow-up period				
No immunosuppressive agents	Reference		Reference	
Prednisolone	9.54 (1.54–182.6)	0.014	11.8 (1.59–242.5)	0.014
Prednisolone+cyclosporine	7.26 (1.29–135.8)	0.022	10.3 (1.59–204.4)	0.012

NOTE: HR, hazard ratio; CI, confidence interval.

Data are the HR, 95% CI, and *P*-value from Cox proportional hazard regression analyses.

Adjusted for baseline characteristics (age, sex, systolic pressure, serum albumin level, serum creatinine level, urinary protein, use of immunosuppressive therapy).

Abbreviations: IMN, idiopathic membranous nephropathy.

After adjusting for clinically relevant factors, older age (adjusted HR = 3.11, 95% CI: 1.45–7.49; *P* = 0.003), prednisolone use (adjusted HR = 11.8, 95% CI: 1.59–242.5; *P* = 0.014), and cyclosporine used in combination with prednisolone (adjusted HR = 10.3, 95% CI: 1.59–204.4; *P* = 0.012) were identified as significant predictors for the development of infection. This suggests that among older IMN patients, immunosuppressive therapy was a more important predictor of infection risk.

Because immunosuppressive therapy use may have been affected by patient age, biasing our estimate of the predictive power of age, we assessed potential associations between patient age and immunosuppressive therapy during the observation period, i.e., the cumulative dose of prednisolone and cyclosporine used during the observation period.

Compared with younger patients, the cumulative dose of prednisolone was lower in elderly patients at 3, 6, and 12 months (*P*<0.001, *P*<0.001, and *P* = 0.002, respectively; **Table S3** **in File S1**). No significant trend between patient age and cyclosporine use emerged at 1, 3, 6, and 12 months (*P* = 0.163, *P* = 0.740, *P* = 0.447, and *P* = 0.387, respectively).

The median times to reach doses of 20, 15, 10, and 7.5 mg/day were shorter in elderly patients than in younger patients (*P* = 0.015, *P* = 0.006, *P*<0.001, and *P*<0.001, respectively; **Table S4 in File S1**). The cumulative doses of prednisolone to reach 20, 15, 10, and 7.5 mg/day were also lower in elderly patients than in younger patients (*P* = 0.006, *P* = 0.005, *P*<0.001, and *P*<0.001, respectively).

These results strongly suggest that the higher incidence of infections in elderly patients was not attributable to the prednisolone dose or to the type of immunosuppressive therapy.

### Predictors of Infections in Patients who Received Immunosuppressive Therapy

In patients who received immunosuppressive therapy (n = 101), we evaluated predictors of infection using clinical data obtained within 1 month from initial immunosuppressive therapy including age, sex, serum creatinine, serum albumin, urinary protein at the time of initiating immunosuppressive therapy, use of immunosuppressive therapy within 1 month after treatment, initial daily dose of prednisolone, and a <25% decrease in proteinuria in the first month after immunosuppressive therapy initiation (**Table 5**).

**Table 5:** Predictors of hospitalization due to infection in patients treated with immunosuppressive therapy.

	Univariate model		Multivariate model	
	HR (95% CI)	*P*-value	HR (95% CI)	*P*-value
Age (per 10 years)	2.71 (1.42–5.55)	0.002	2.80 (1.20–7.83)	0.016
Male (versus female)	1.16 (0.34–5.21)	0.827	1.65 (0.39–8.46)	0.500
Serum albumin (per 1.0 g/dL)	1.12 (0.46–2.55)	0.792	2.15 (0.64–7.99)	0.217
Serum creatinine (per 1.0 mg/dL)	4.15 (1.42–9.53)	0.013	1.15 (0.31–3.88)	0.830
Urinary protein excretion (per 1.0 g/day)	0.92 (0.73–1.09)	0.374	0.94 (0.70–1.19)	0.633
Immunosuppressive treatment within 1 monthafter kidney biopsy				
Prednisolone	Reference		Reference	
Prednisolone+cyclosporine	1.45 (0.46–4.89)	0.524	3.22 (0.74–16.9)	0.119
Initial dose of PSL/mg/day	0.97 (0.92–1.01)	0.162	1.00 (0.94–1.07)	0.943
25% decrease of proteinuria within 1 monthafter initial immunosuppressive therapy	5.78 (1.67–26.4)	0.005	7.27 (1.74–37.7)	0.007

NOTE: HR, hazard ratio; CI, confidence interval.

Data are the HR, 95% CI, and *P*-value from Cox proportional hazard regression analyses.

This analysis is based on data from 100 patients because the decrease rate of proteinuria was missing for one patient.

Adjusted for baseline characteristics (age, sex, systolic/diastolic pressure, serum albumin level, serum creatinine level, urinary protein, use of immunosuppressive therapy, initial dose of PSL (mg)/day, 25% decrease of proteinuria within 1 month after initial immunosuppressive therapy).

Abbreviations: IMN, idiopathic membranous nephropathy.

Univariate CPH models showed that age, serum creatinine, and a <25% decrease in proteinuria in the first month after immunosuppressive therapy initiation were significant risk factors for developing infection. A multivariate CPH model adjusted for age, sex, creatinine, proteinuria at the time of initiating immunosuppressive therapy, use of immunosuppressive therapy within 1 month after starting therapy, and initial daily dose of prednisolone, was then applied. In the multivariate analysis, older age (adjusted HR = 2.80, 95% CI: 1.20–7.83; *P* = 0.016) and a <25% decrease in proteinuria in the first month after immunosuppressive therapy initiation (adjusted HR = 7.27, 95% CI: 1.74–37.7; *P* = 0.007) were identified as significant risk factors. The −2 log likelihood values with all explanatory variables were 39.32, 39.93, and 39.96, respectively, for Cox models that included a 25% decrease, 50% decrease, and 75% decrease in proteinuria in the first month after immunosuppressive therapy initiation. Because the degrees of freedom were the same for these three models, these results indicate that the Cox model that included a 25% decrease in proteinuria was the best fit to the observed infection events.

We further stratified the decreasing rate of proteinuria in the first month after the initial multivariate analysis, and found that patients with a poor response to initial therapy had a nearly linear high risk for infection (*P* = 0.006, **Table 6**).

**Table 6:** Predictors of hospitalization due to infection.

	Univariate model		Multivariate model	
	HR (95% CI)	*P*-value	HR (95% CI)	*P*-value
Decrease of proteinuria within1 month (%)				
≥50% decrease	Reference		Reference	
25–50% decrease	0.60 (0.03–3.17)	0.609	2.27 (0.10–25.3)	0.535
0–25% decrease	7.98 (1.56–57.6)	0.014	6.52 (0.99–54.3)	0.051
Exacerbation	6.05 (1.18–43.6)	0.031	14.4 (1.87–145.6)	0.011
Test for trend		0.011		0.006

NOTE: HR, hazard ratio; CI, confidence interval.

Data are the HR, 95% CI, and *P*-value from Cox proportional hazard regression analyses. This analysis is based on data from 100 patients because the decrease rate of proteinuria was missing for one patient. Adjusted for baseline characteristics (age, sex, systolic/diastolic pressure, serum albumin level, serum creatinine level, urinary protein, use of immunosuppressive therapy, initial dose of PSL (mg)/day, 25% decrease of proteinuria within 1 month after initial immunosuppressive therapy).

Because the decrease in proteinuria in the first month after initial immunosuppressive therapy may have affected subsequent treatment, resulting in bias, we evaluated the associations between proteinuria decreases (<25% vs. ≥25%) and immunosuppressive therapy during the observation period, namely, the cumulative dose of prednisolone and immunosuppressive agent use during the observation period (**Table S5 in File S1**). There were no significant differences between patients achieving <25% and ≥25% decreases in proteinuria with respect to the initial prednisolone dose, cyclosporine use in the first month, and the cumulative dose of prednisolone at 3, 6, and 12 months (*P* = 0.164, *P* = 0.553, and *P* = 0.678, respectively). No significant trend was between age and cyclosporine use was observed at 3, 6, and 12 months (*P* = 0.526, *P* = 0.139, and *P* = 0.675, respectively).

The median times to reach 20, 15, 10, and 7.5 mg/day doses did not between the two groups (*P* = 0.143, *P* = 0.349, *P* = 0.431, and *P* = 0.650, respectively; **Table S6 in File S1**). The cumulative doses of prednisolone to reach 20, 15, 10, and 7.5 mg/day were not different between these two groups (*P* = 0.138, *P* = 0.277, *P* = 0.958, and *P* = 0.871, respectively).

These results suggest that patients with a <25% decrease in proteinuria at 1 month did not subsequently receive a higher dose of prednisolone.

## Discussion

In the current study, we investigated the clinical characteristics and outcomes of older IMN patients as compared with younger IMN patients. Furthermore, this is the first study to have examined predictors of infection in a large cohort of IMN patients.

Consistent with a previous study [2], we found that the remission rate, a 30% decline in eGFR, and the rate of decline in renal function after the onset of MN were similar in older and younger IMN patients. However, our elderly patients may have died before developing a 30% decline in eGFR because the relationship between age and renal dysfunction may have been underestimated.

Our study included older patients than previously published cohort studies. Nevertheless, both remission and renal survival rates in the present study were higher than those reported by Ponticelli et al. and Jha et al. [5], [6]. This indicates that Japanese patients achieve a benign course compared to patients in other countries.

Unlike previous studies [4], [10], we observed that compared to younger patients, older patients presented with more severe clinical findings such as lower serum albumin levels, leg edema, and pleural effusion. In elderly patients, edema is often attributed to heart failure or venous insufficiency of the lower limbs. Edema occurs more readily in elderly patients because of reduced elasticity of the skin and interstitial tissues. Thus, edema may coincide with higher serum albumin levels in the elderly [14].

With regard to immunosuppressive therapy, the recently published Kidney Disease Improving Global Outcomes (KDIGO) guidelines for IMN recommended a restrictive treatment strategy for IMN patients [15]. According to these guidelines, initial therapy should be started only if proteinuria is persistently >4 g/day after 6 months of conservative therapy and does not show a tendency to decline, if the serum creatinine concentration increases by >30%, or if severe, disabling, or life-threatening symptoms related to nephrotic syndrome are present. However, the KDIGO recommendations were based on studies that included only a small number of elderly patients. Therefore, the optimal immunosuppressive regimen for elderly IMN patients remains unclear.

Two retrospective studies involving 115 patients older than 60–65 years found little evidence for the benefits of glucocorticoid monotherapy along with a higher incidence of adverse effects such as infection, peptic ulcer, and gastrointestinal disturbances [4], [16]. Furthermore, many patients with mild-to-moderate disease achieve a spontaneous remission [17]. These studies therefore suggest that in older patients, immunosuppressive therapy should be considered only for those who are at high risk for progression and only after maximum conservative therapy has failed [11], [18], [19]. However, these studies provide no information regarding clinical presentation, including the prevalence of pleural effusion or the corticosteroid dose used during the follow-up period, which are important points for consideration in the elderly.

In the present study, the time from kidney biopsy to immunosuppressive therapy initiation was shorter than that recommended in the KDIGO guidelines [15]. However, our results show that elderly patients had more clinically severe symptoms than younger patients. Thus, Japanese doctors may consider that in patients with clinically severe symptoms, immunosuppressive therapy should be started as soon as possible, exhibiting different practice patterns than those observed in other countries.

As in previous studies, our elderly patients were significantly predisposed to infection. The infection incidence in our cohort did not greatly differ from those observed previously [20], [21]. We found that the higher incidence of infection among elderly patients was unlikely to be due to more intensive immunosuppressive therapy because the cumulative corticosteroid dose in this age group was lower than that in younger patients. Elderly IMN patients were found to be at a higher risk for infection than younger patients.

Interestingly, we found that a <25% decrease in proteinuria at 1 month after starting immunosuppressive therapy was a significant predictor of infection. Furthermore, the higher incidence of infection associated with a <25% decrease in proteinuria in the first month after initial immunosuppressive therapy was not attributable to the prednisolone dose or the type of immunosuppressive therapy. These results suggest that poor responders after the first month of immunosuppressive therapy are vulnerable to the development of infection.

Therefore, it might be well advised to taper prednisolone more quickly among patients with a poor response to 1 month of initial immunosuppressive therapy.

However, it has also been postulated that severe nephrotic syndrome is associated with an immunologic deficit that predisposes to the development of infection. Susceptibility to bacterial infections in patients with a nephrotic syndrome has been attributed to decreased levels of IgG and the alternative complement factor B [11]. Compared with younger patients, older IMN patients had more severe symptoms of nephrotic syndrome, which may contribute to subsequent infections after the initiation of immunosuppressive therapy. Admittedly, this is a complicated subject, and it is unlikely that this retrospective analysis can address all the issues necessary to reach a conclusion whether the treatment should be intensified or not.

Our study had several limitations. First, our patients may not be representative of IMN patients in other countries. Therefore, we advise caution when interpreting and generalizing our results. Second, due to the retrospective nature of this study, the criteria used to select patients’ therapeutic regimens are unknown. Selected regimens may vary across different centers, eras, or physicians. These potential biases should be included in the analyses. In actuality, we could carry out only patient-level analysis adjusted for clinically relevant factors but could not carry out a facility-level analysis to reduce confounding by indication concerning therapy selection. This is because the present study included as many as 10 nephrology centers, some of which treated a patient number too small to evaluate using Cox proportional hazard models. Furthermore, it was difficult to add era as a covariate in our models. Third, our practice patterns are different from those recommended by the KDIGO guidelines. Namely, we often use cyclosporine in combination with corticosteroids for the first-line treatment of IMN patients. The 2012 KDIGO clinical practice guidelines for IMN recommend a cytotoxic agent (cyclophosphamide) for patients at high risk of progression [15]. However, no patients in our study were treated with cyclophosphamide. Therefore, our results should be interpreted carefully. Fourth, because the time from IMN diagnosis to immunosuppressive therapy initiation was relatively short, we did not evaluate whether nephrotic syndrome itself would predispose to infection by observing patients without immunosuppressive therapy.

Allowing for these methodological issues, our study has several advantages. It is one of the largest multicenter adult Japanese MN cohorts ever reported. Additionally, to the best of our knowledge, this is the first study to evaluate predictors of infection in IMN patients.

In conclusion, our retrospective cohort study of IMN patients showed that elderly patients were similar to younger patients in terms of renal outcomes. However, the use of immunosuppressive therapy and poor response to initial immunosuppressive therapy were significant predictors of infection among elderly IMN patients. Therefore, care should be taken when selecting a treatment strategy for these patients.

## Supporting Information

Figure S1 **The course of proteinuria during the follow-up period (comparison of the three age categories).**
(TIF)

Figure S2 **The course of eGFR during the follow-up period (comparison of the three age categories).**
(TIF)

File S1 **Table S1 in File S1,** Baseline characteristics of 171 IMN patients: comparison of patients in different treatment groups. **Table S2,** Outcomes of 171 IMN patients: comparison of patients in different treatment groups. **Table S3,** Immunosuppressive treatment during the observation period (comparison of the three age categories). **Table S4,** Duration and cumulative dose of prednisolone (comparison of the three age categories). **Table S5,** Immunosuppressive treatment during the observation period (comparison of the decrease in proteinuria (<25% vs. ≥25%) in the first month after starting immunosuppressive therapy). **Table S6,** Duration and cumulative dose of prednisolone (comparison of the decrease in proteinuria (<25% vs. ≥25%) in the first month after starting immunosuppressive therapy).
(DOC)

File S2 **Table S7,** The anonymous data set of 171 patients with IMN.
(XLSX)
