# Supplementary figures and images for: Patient Age and the Prognosis of Idiopathic Membranous Nephropathy
Source: PLoS One. 2014 Oct 20;9(10):e110376. doi: 10.1371/journal.pone.0110376 (PMC4203783; doi:10.1371/journal.pone.0110376)

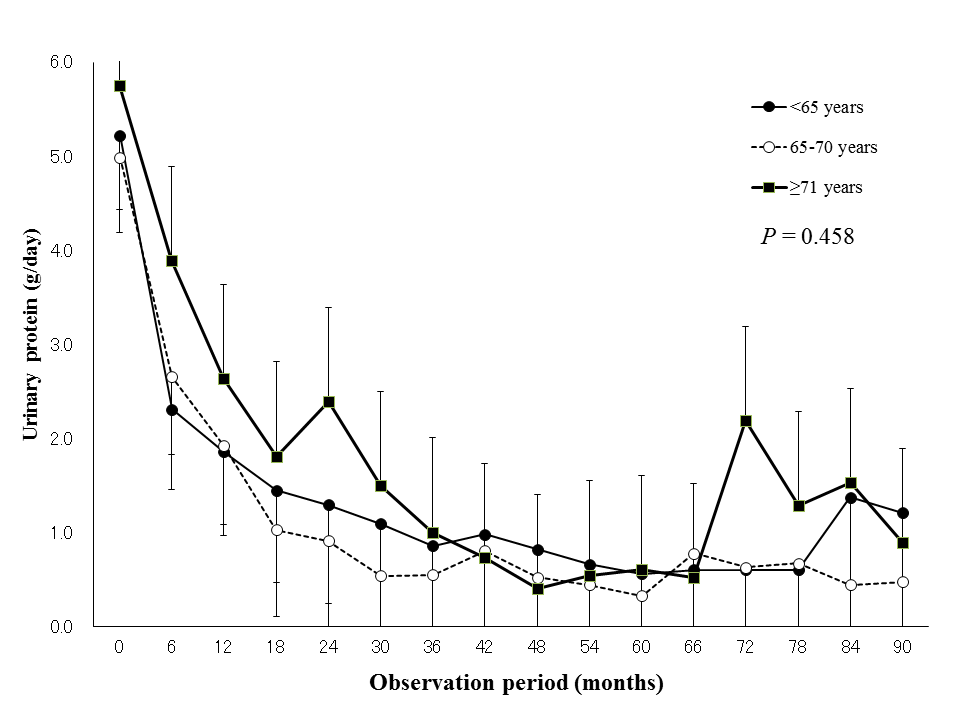

Supplement: Figure S1 — The course of proteinuria during the follow-up period (comparison of the three age categories). (TIF) [file pone.0110376.s001.tif]

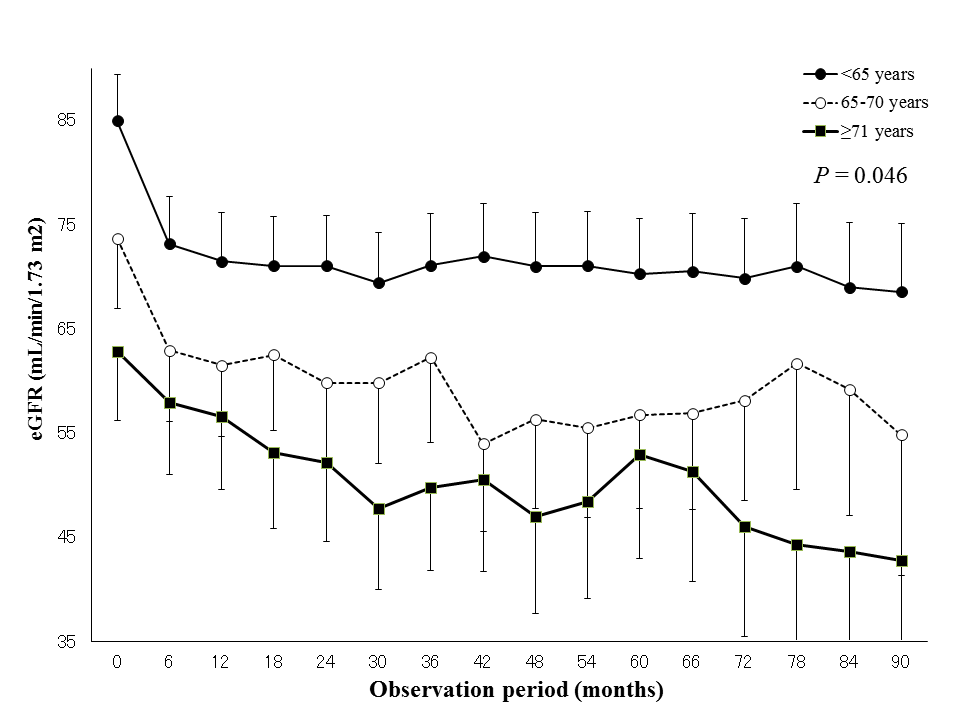

Supplement: Figure S2 — The course of eGFR during the follow-up period (comparison of the three age categories). (TIF) [file pone.0110376.s002.tif]
