# Supplementary material for: Patient Age and the Prognosis of Idiopathic Membranous Nephropathy
Source: PLoS One. 2014 Oct 20;9(10):e110376. doi: 10.1371/journal.pone.0110376 (PMC4203783; doi:10.1371/journal.pone.0110376)
Supplement: File S1 — Table S1 in File S1, Baseline characteristics of 171 IMN patients: comparison of patients in different treatment groups. Table S2, Outcomes of 171 IMN patients: comparison of patients in different treatment groups. Table S3, Immunosuppressive treatment during the observation period (comparison of the three age categories). Table S4, Duration and cumulative dose of prednisolone (comparison of the three age categories). Table S5, Immunosuppressive treatment during the observation period (comparison of the decrease in proteinuria (<25% vs. ≥25%) in the first month after starting immunosuppressive therapy). Table S6, Duration and cumulative dose of prednisolone (comparison of the decrease in proteinuria (<25% vs. ≥25%) in the first month after starting immunosuppressive therapy). (DOC) [file pone.0110376.s003.doc]

**Table S1.** Baseline characteristics of 171 IMN patients: comparison of patients with different treatments

|  | Supportive therapy | Prednisolone monotherapy | Prednisolone + Cyclosporine | *P*-value |
| --- | --- | --- | --- | --- |
| Number | 70 | 35 | 66 |  |
| Baseline characteristics |  |  |  |  |
| Age (years) | 64 (59–70) | 65 (54–69) | 64 (57–70) | 0.922 |
| Male [n (%)] | 46 (65.7) | 22 (62.9) | 50 (75.8) | 0.304 |
| Body mass index (kg/m^2^) | 22.8 (21.2–25.1) | 23.4 (20.9–24.9) | 23.4 (22.0–25.9) | 0.404 |
| Systolic blood pressure (mmHg) | 132 (121–142) | 132 (126–146) | 129 (118–150) | 0.753 |
| Diastolic blood pressure (mmHg) | 76 (70–82) | 80 (70–88) | 78 (70–85) | 0.544 |
| Serum creatinine (mg/dL) | 0.8 (0.7–1.0) | 0.8 (0.7–1.1) | 0.8 (0.7–1.0) | 0.625 |
| eGFR (mL/min/1.73 m^2^) | 77 (60–93) | 80 (53–93) | 74 (60–88) | 0.692 |
| Serum albumin (g/dL) | 3.2 (2.5–3.7) | 2.4 (1.9–3.2) | 2.2 (1.8–2.7) | <0.001 |
| Urinary protein (g/day) | 3.4 (1.9–5.5) | 5.0 (3.5–6.8) | 5.2 (3.6–8.3) | <0.001 |
| Urinary protein >3.5 (g/day) [n (%)] | 34 (48.6) | 27 (77.1) | 53 (80.3) | <0.001 |
| Total cholesterol (mg/dL) | 260 (218–308) | 304 (241–415) | 333 (265–397) | <0.001 |
| Leg edema [n (%)] | 43 (61.4) | 30 (85.7) | 58 (87.9) | <0.001 |
| Pleural effusion [n (%)] | 7 (10.0) | 11 (31.4) | 15 (22.7) | 0.021 |
| Treatment |  |  |  |  |
| ACE inhibitor or ARB therapy [n (%)] | 67 (95.7) | 32 (91.4) | 57 (86.4) | 0.156 |
| Observational period (months) | 45 (15–74) | 29 (15–88) | 35 (16–63) | 0.757 |

NOTE: Median (interquartile range), Conversion factors for units: SCr in mg/dL to μmol/L, ×88.4; eGFR (mL/min/1.73 m2) = 194 × Scr^-1.094^× Age^-0.287^ × 0.739 (if female), total cholesterol in mg/dL to mmol/L, ×0.02586.

Abbreviations: IMN, idiopathic membranous nephropathy; eGFR, estimated glomerular filtration rate; ACE inhibitor/ARB, angiotensin-converting enzyme inhibitor/angiotensin receptor blocker.

**Table S2.** Outcomes of 171 IMN patients: comparison of patients with different treatments

|  | Supportive therapy | Prednisolone monotherapy | Prednisolone + Cyclosporine | P-value |
| --- | --- | --- | --- | --- |
| Number | 70 | 35 | 66 |  |
| 30% reduction in eGFR [n (%)] | 17 (18.9) | 9 (22.5) | 11 (26.8) | 0.591 |
| Decline in eGFR (mL/min per 1.73m^2^ per year) | 2.18 (-0.25–5.78) | 2.77 (-0.05–6.91) | 3.76 (0.49–9.73) | 0.300 |
| ESRD [n (%)] | 0 (0.0) | 2 (5.7) | 0 (0.0) | 0.011 |
| Death [n (%)] | 2 (2.9) | 6 (17.1) | 3 (4.6) | 0.014 |
| Death due to infection [n (%)] | 1 (1.4) | 4 (11.4) | 2 (3.0) | 0.044 |
| Hospitalization due to infection [n (%)] | 1 (1.4) | 5 (14.3) | 7 (10.6) | 0.032 |
| Hospitalization due to cardiovascular disease [n (%)] | 2 (2.9) | 0 (0.0) | 1 (1.5) | 0.565 |
| Venous thrombotic events [n (%)] | 0 (0.0) | 0 (0.0) | 0 (0.0) | 1.000 |
| Malignancy [n (%)] | 3 (4.3) | 1 (2.9) | 1 (1.5) | 0.632 |
| Steroid psychosis [n (%)] | 0 (0.0) | 2 (5.7) | 1 (1.5) | 0.108 |
| Use of antidiabetic agents [n (%)] | 2 (2.9) | 3 (8.6) | 8 (12.1) | 0.122 |
| Aseptic osteonecrosis with surgical treatment [n (%)] | 0 (0.0) | 0 (0.0) | 0 (0.0) | 1.000 |
| Remission |  |  |  |  |
| Complete remission [n (%)] | 34 (48.6) | 25 (71.4) | 44 (66.7) | 0.031 |
| Partial remission [n (%)] | 61 (87.1) | 29 (82.9) | 60 (90.9) | 0.493 |
| Relapse [n (%)] | 7 (14.0) | 3 (11.5) | 16 (28.6) | 0.086 |

NOTE: Median (interquartile range), Conversion factors for units: SCr in mg/dL to μmol/L, ×88.4; eGFR (mL/min/1.73 m2) = 194 × Scr^-1.094^× Age^-0.287^ × 0.739 (if female), total cholesterol in mg/dL to mmol/L, ×0.02586.

Abbreviations: IMN, idiopathic membranous nephropathy; eGFR, estimated glomerular filtration rate; ESRD, end-stage renal disease.

**Table S3.** Immunosuppressive treatment during the observation period

|  | Baseline age (years) | | | *P*-value |
| --- | --- | --- | --- | --- |
|  | <65 years | 65–70 years | ≥71 years |  |
| Total number | 54 | 23 | 24 |  |
| Initial therapy |  |  |  |  |
| Prednisolone initial dose ［mg/day］ | 40 (25–46) | 30 (20–40) | 30 (21–38) | 0.044 |
| Use of cyclosporine at 1^st^ month [n (%)] | 27 (50.0) | 11 (47.8) | 13 (54.2) | 0.905 |
| At 3rd month |  |  |  |  |
| Number at risk (n) | 53 | 23 | 20 |  |
| Cumulative dose of prednisolone (g) | 2.7 (1.9–3.5) | 2.3 (1.7–2.6) | 2.0 (1.3–2.3) | <0.001 |
| Use of cyclosporine [n (%)] | 30 (56.7) | 13 (56.5) | 13 (65.0) | 0.864 |
| At 6th month |  |  |  |  |
| Number at risk (n) | 52 | 21 | 18 |  |
| Cumulative dose of prednisolone (g) | 3.9 (3.0–5.0) | 3.2 (2.6–4.0) | 3.0 (2.2–3.4) | <0.001 |
| Use of cyclosporine [n (%)] | 32 (61.5) | 10 (43.5) | 14 (77.8) | 0.660 |
| At 1st year |  |  |  |  |
| Number at risk (n) | 41 | 19 | 13 |  |
| Cumulative dose of prednisolone (g) | 5.9 (4.2–7.1) | 4.7 (3.5–5.6) | 4.1 (3.2–4.6) | 0.002 |
| Use of cyclosporine [n (%)] | 29 (70.7) | 8 (42.1) | 9 (69.2) | 0.228 |

Median (interquartile range)

**Table S4.** Duration and cumulative dose of prednisolone

|  | Decrease of proteinuria at 1st month | | | *P*-value |
| --- | --- | --- | --- | --- |
|  | <65 years | 65–70 years | ≥71 years |  |
| Total number | 54 | 23 | 24 |  |
| PSL 20 mg/day |  |  |  |  |
| Number at risk (n) | 53 | 23 | 24 |  |
| Time to reduce to 20 mg/day, months | 3.0 (1.8–5.0) | 2.0 (0.0–3.3) | 2.0 (0.1–3.0) | 0.015 |
| Cumulative dose to reach 20 mg/day of prednisolone (g) | 2.1 (0.8–3.9) | 1.2 (0.0–2.6) | 1.0 (0.1–1.7) | 0.006 |
| PSL 15 mg/day |  |  |  |  |
| Number at risk (n) | 53 | 23 | 22 |  |
| Time to reduce to 15 mg/day, months | 4.1 (3.0–6.0) | 4.0 (3.0–5.0) | 3.0 (0.6–4.0) | 0.006 |
| Cumulative dose to reach 15 mg/day of prednisolone (g) | 2.9 (1.7–4.4) | 2.3 (1.2–3.2) | 1.7 (0.4–2.6) | 0.005 |
| PSL 10 mg/day |  |  |  |  |
| Number at risk (n) | 51 | 22 | 21 |  |
| Time to reduce to 10 mg/day, months | 7.0 (5.0–9.2) | 5.0 (4.0–8.0) | 4.0 (2.3–6.0) | <0.001 |
| Cumulative dose to reach 10 mg/day of prednisolone (g) | 4.1 (2.7–5.6) | 3.1 (2.1–4.7) | 2.5 (1.2–3.4) | <0.001 |
| PSL 7.5 mg/day |  |  |  |  |
| Number at risk (n) | 47 | 22 | 21 |  |
| Time to reduce to 7.5 mg/day, months | 8.5 (6.8–16.3) | 7.0 (5.5–12.0) | 6.5 (3.9–8.8) | 0.003 |
| Cumulative dose to reach 7.5 mg/day of prednisolone (g) | 4.5 (3.5–9.0) | 3.4 (2.6–5.0) | 3.3 (2.0–4.4) | <0.001 |

Median (interquartile range)

**Table S5.** Immunosuppressive treatment during the observation period

|  | Decrease of proteinuria at 1st month | | *P*-value |
| --- | --- | --- | --- |
|  | <25% | ≥25% |  |
| Total number | 35 | 65 |  |
| Initial therapy |  |  |  |
| Prednisolone initial dose [mg/day] | 30 (20–40) | 35 (25–40) | 0.180 |
| Use of cyclosporine at the 1^st^ month [n (%)] | 14 (40.0) | 37 (56.9) | 0.142 |
| At 3rd month |  |  |  |
| Number at risk (n) | 31 | 64 |  |
| Cumulative dose of prednisolone (g) | 2.3 (1.4–2.8) | 2.4 (1.8–3.2) | 0.164 |
| Use of cyclosporine [n (%)] | 18 (58.1) | 38 (59.4) | 0.526 |
| At 6th month |  |  |  |
| Number at risk (n) | 28 | 62 |  |
| Cumulative dose of prednisolone (g) | 3.3 (2.7–4.1) | 3.6 (2.8–4.8) | 0.553 |
| Use of cyclosporine [n (%)] | 16 (57.1) | 40 (64.5) | 0.139 |
| At 1st year |  |  |  |
| Number at risk (n) | 21 | 51 |  |
| Cumulative dose of prednisolone (g) | 5.0 (4.0–6.3) | 4.7 (2.8–6.6) | 0.678 |
| Use of cyclosporine [n (%)] | 15 (71.4) | 31 (60.8) | 0.675 |

Median (interquartile range)

**Table S6.** Duration and cumulative dose of prednisolone

|  | Decrease of proteinuria at 1st month | | *P*-value |
| --- | --- | --- | --- |
|  | <25% | ≥25% |  |
| Total number | 35 | 65 |  |
| PSL 20 mg/day |  |  |  |
| Number at risk (n) | 35 | 64 |  |
| Time to reduce to 20 mg/day, months | 2.5 (0.0–3) | 3.0 (0.7–4.0) | 0.143 |
| Cumulative dose to reach 20 mg/day of prednisolone (g) | 1.4 (0.0–2.3) | 1.7 (0.4–3.5) | 0.138 |
| PSL 15 mg/day |  |  |  |
| Number at risk (n) | 33 | 64 |  |
| Time to reduce to 15 mg/day, months | 4.0 (1.1–5.0) | 4.0 (3.0–5.8) | 0.349 |
| Cumulative dose to reach 15 mg/day of prednisolone (g) | 2.3 (1.1–3.3) | 2.7 (1.5–4.1) | 0.277 |
| PSL 10 mg/day |  |  |  |
| Number at risk (n) | 31 | 62 |  |
| Time to reduce to 10 mg/day, months | 5.0 (4.0–8.0) | 6.0 (4.5–8.0) | 0.431 |
| Cumulative dose to reach 10 mg/day of prednisolone (g) | 3.5 (2.5–4.6) | 3.3 (2.4–5.1) | 0.958 |
| PSL 7.5 mg/day |  |  |  |
| Number at risk (n) | 29 | 60 |  |
| Time to reduce to 7.5 mg/day, months | 8.0 (5.0–14.0) | 8.0 (6.0–12.0) | 0.650 |
| Cumulative dose to reach 7.5 mg/day of prednisolone (g) | 4.4 (2.9–6.2) | 4.1 (2.9–5.7) | 0.871 |

Median (interquartile range)
